# Evaluation of Non-Invasive Multispectral Imaging as a Tool for Measuring the Effect of Systemic Therapy in Kaposi Sarcoma

**DOI:** 10.1371/journal.pone.0083887

**Published:** 2013-12-27

**Authors:** Jana M. Kainerstorfer, Mark N. Polizzotto, Thomas S. Uldrick, Rafa Rahman, Moinuddin Hassan, Laleh Najafizadeh, Yasaman Ardeshirpour, Kathleen M. Wyvill, Karen Aleman, Paul D. Smith, Robert Yarchoan, Amir H. Gandjbakhche

**Affiliations:** 1 Section on Analytical and Functional Biophotonics, Eunice Kennedy Shriver National Institute of Child Health and Human Development, National Institutes of Health, Bethesda, Maryland, United States of America; 2 HIV and AIDS Malignancy Branch, National Cancer Institute, National Institutes of Health, Bethesda, Maryland, United States of America; 3 Biomedical Instrumentation and Multiscale Imaging Section, Laboratory of Cellular Imaging and Macromolecular Biophysics, National Institute of Biomedical Imaging and Bioengineering, National Institutes of Health, Bethesda, Maryland, United States of America; University of Navarra, Spain

## Abstract

Diffuse multi-spectral imaging has been evaluated as a potential non-invasive marker of tumor response. Multi-spectral images of Kaposi sarcoma skin lesions were taken over the course of treatment, and blood volume and oxygenation concentration maps were obtained through principal component analysis (PCA) of the data. These images were compared with clinical and pathological responses determined by conventional means. We demonstrate that cutaneous lesions have increased blood volume concentration and that changes in this parameter are a reliable indicator of treatment efficacy, differentiating responders and non-responders. Blood volume decreased by at least 20% in all lesions that responded by clinical criteria and increased in the two lesions that did not respond clinically. Responses as assessed by multi-spectral imaging also generally correlated with overall patient clinical response assessment, were often detectable earlier in the course of therapy, and are less subject to observer variability than conventional clinical assessment. Tissue oxygenation was more variable, with lesions often showing decreased oxygenation in the center surrounded by a zone of increased oxygenation. This technique could potentially be a clinically useful supplement to existing response assessment in KS, providing an early, quantitative, and non-invasive marker of treatment effect.

## Introduction

Kaposi sarcoma (KS) is a multicentric angioproliferative tumor caused by Kaposi sarcoma-associated herpes virus, also known as human herpes virus 8 (KSHV/HHV-8) [1], [2], [3], [4], [5]. It is most common in patients with immunodeficiencies, including HIV infection and iatrogenically following organ transplantation, but also occurs in immunocompetent individuals [6], [7]. Especially in the setting of HIV/AIDS, KS can be associated with significant morbidity and mortality. Clinically, KS is characterized by the development of cutaneous lesions, and in severe cases may also involve the internal viscera and lymphatics. Early lesions may be flat, while advanced lesions may be nodular and ulcerate. Lesions are comprised of spindle cells and infiltrating mononuclear cells around leaky vascular slits. Red blood cells and their breakdown products, including hemosiderin, in this abnormal vasculature give KS its distinctive red, purple or brown coloration. Dysregulated angiogenesis is a hallmark of KS, with the characteristic vascularity resulting from pro-angiogenic factors produced by the KSHV-infected tumor spindle cells, including induction of basic fibroblast growth factor (bFGF) and vascular endothelial growth factor (VEGF) [8], [9].

Assessment of the response of KS lesions to therapy is challenging. In particular, assessment of lesion size can be misleading as nodular lesions may enlarge in area even as they flatten and respond to therapy. Also, discordant responses (with some lesions improving while others progress) are not uncommon [10]. Responding lesions may also leave residual hemosiderin deposits in the skin, whose brown coloration can be difficult to distinguish visually from active disease [10]. Common oncologic definitions of response are difficult to apply to multifocal tumors such as KS. Recognizing these issues, in an effort to standardize the evaluation of therapy for KS, the AIDS Clinical Trial Group (ACTG) Oncology Committee developed a set of response definitions for KS [11]. These incorporate counts of lesion numbers and nodularity, assessment of the size of (usually five) ‘representative’ lesions, and assessment of lesion coloration. Assessment of responses requires detailed evaluations of multiple skin lesions, which are time intensive, require skilled practitioners, and suffer from inter-observer variability.

These issues, together with accessibility and its vascular nature, make cutaneous KS an excellent tumor type for exploring the feasibility of employing non-invasive imaging techniques for the evaluation of tumor vasculature and angiogenesis. Diffuse multispectral imaging of the skin and image reconstruction of skin chromophores have found their application in the clinic, successfully assessing parameters for healthy individuals as well as for those with cutaneous diseases [12], [13], [14], [15], [16], [17], [18], [19]. Acquiring several images of the lesion at different wavelengths in the near infrared spectrum, together with employing an analytical skin model for fitting the data, allows for extracting and mapping of the spatial distribution of blood volume and oxygenation concentrations [16], [20], [21]. The disadvantage of this method lies in its computationally intensive data post processing, which makes real time conclusions difficult. Principal component analysis (PCA) found applications in fields such as face recognition [22], [23], and image compression [24], and is a common technique for finding patterns in highly dimensional data [25]. The goal of PCA is to reveal the data that best explain the variance in the data. The advantages of PCA are its computation speed, which is in the order of seconds per image, as well as its model independence. Using PCA for extracting blood and melanin values has been proposed [26], showing that skin color in digital RGB images can be described by the first two principal components. PCA and independent component analysis (ICA) have also been applied to RGB imaging data for extraction of blood and melanin values in vitiligo lesions to qualitatively evaluate the skin re-pigmentation progression [27], [28]. Our group has shown previously [29] that PCA applied to multispectral images from the skin in the wavelength range between 750 nm and 850 nm can be used for mapping the blood volume and blood oxygenation, where the first eigenvector (first principal component) describes the blood volume and the second eigenvector corresponds to the blood oxygenation. This description was found to qualitatively match the temporal behavior and spatial distribution of reconstruction results, using a two-layered analytical skin model.

Here we evaluated multi-spectral imaging of cutaneous KS lesions, using PCA to assess blood volume and oxygenation. We hypothesized that reductions in lesion blood volume would be detectable by multi-spectral imaging and would correlate with treatment outcome as assessed by clinical and, when appropriate, pathological criteria. Blood volume and oxygenation were evaluated over the course of treatment, allowing further comparison of the time at which responses were detected by multispectral imaging compared with conventional measures.

## Patients and Methods

### Ethics Statement

The human subject examined was recruited from a large imaging study using non-invasive multi-spectral imaging. The treatment and imaging protocols were each approved by the NCI Institutional Review Board. All subjects gave written informed consent.

### Clinical Population

Patients with cutaneous KS confirmed by histopathology and undergoing therapy for KS on protocols within the clinical program of the HIV and AIDS Malignancy Branch (HAMB), National Cancer Institute (NCI) were studied. These studies utilized bevacizumab antiangiogenic therapy alone in one study (one subject)[30], or bevacizumab in combination with cytotoxic chemotherapy with liposomal doxorubicin followed by antiangiogenic therapy alone (remaining subjects) (ClinicalTrials.gov Identifier NCT00923936). Diffuse multispectral analysis was performed on a ‘target’ KS lesion at baseline and then approximately every 12 weeks during therapy. Patients were all followed until 80 weeks of treatment unless complete responses occurred sooner, with the exception of patient #6 who left the treatment study at the time of disease progression at week 21.

### Overall and Target Lesion Clinical KS Response Assessment

A modification of ACTG response definitions was used to assess overall clinical responses [7], [11], [30]. Briefly, prior to commencement of therapy, five marker lesions representative of the patient’s disease and, where possible, located on separate areas of the body, were selected for clinical response assessment. Marker lesions could not have been previously treated with local therapies such as radiation therapy or intra-lesional injections. In addition, the overall extent of cutaneous KS was assessed by counting the total number of lesions (for subjects with fewer than 50 lesions) or the total number of lesions on up to three representative areas of the body (for subjects with more than 50 lesions), and recording their color and whether or not they were nodular. Changes in the area (the sum product of the diameters) of the marker lesions, lesion number, and lesion nodularity were then assessed regularly to assess the clinical response to therapy. In short, an overall partial response (PR) required a 50% decrease in the number of lesions, or the sum product of the diameters of the marker lesions, or the number of nodular lesions, without meeting any parameters of progressive disease. A complete response (CR) required clinical resolution of all lesions (except perhaps for some residual pigmentation) and tumor-associated edema with biopsy conformation of a cutaneous lesion that was previously involved. Progressive disease was considered a 25% increase in the same parameters (number of lesions, area, or nodular lesions).

For each patient, a single ‘target’ lesion was selected for imaging. The target lesion in each case was one of the marker lesions, chosen for accessibility to the multi-spectral instrument (usually on the arm). This lesion was imaged before treatment, approximately every 12 weeks during therapy, and again following completion of therapy. A clinical response in the target lesion was defined as either flattening of a previous nodular lesion, a 50% or greater decrease in lesion area, or the lesion resolving completely (again except for perhaps some residual pigmentation). Those cases in which a lesion as well as all non-target lesions were felt to have resolved completely, a biopsy of a non-target lesion was required to obtain pathologic confirmation that there was no residual tumor. If the patient was thereby designated as having a CR, it was assumed that the target lesion similarly resolved.

### Non-invasive Imaging Instrumentation

**Figure 1:**
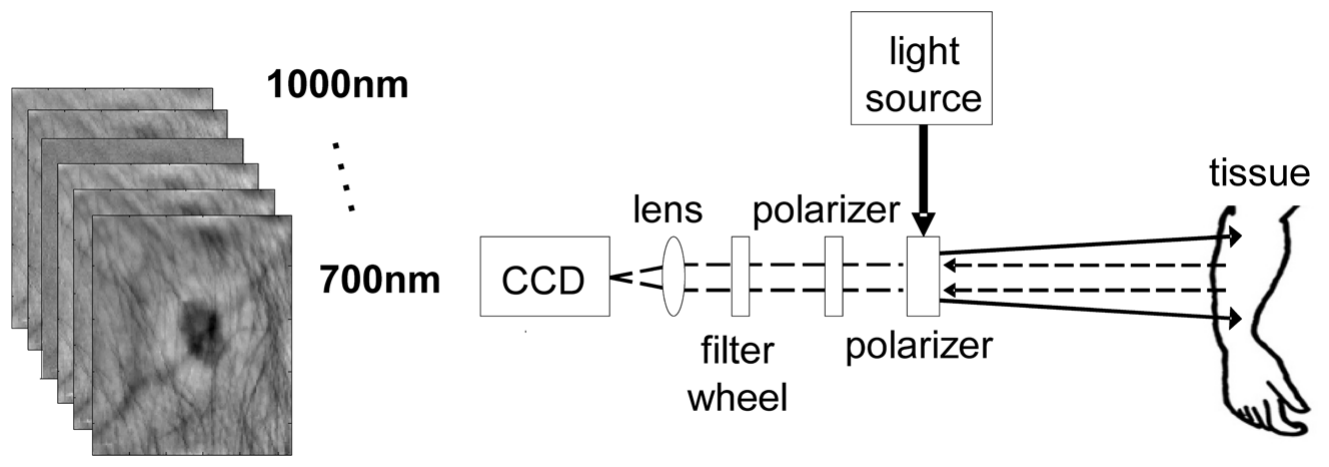
Multi-spectral instrument. Linearly polarized light is projected onto the skin and diffuse reflectance images are captured at the CCD camera after passing narrow band filters for wavelength selection.

Diffuse multispectral systems acquire 2D images of specific wavelengths. A schematic of the instrument, described elsewhere[16], can be seen in Fig. 1. A broadband linearly polarized light source (halogen 150 W, Techniquip, Pleasanton, California) is used for illumination with a fiber waveguide. Reflected light passes a second polarizer, with its orientation perpendicular to the incident polarization plane. Thus, only cross-polarized light passes the second polarizer, which carries information about deeper tissue layers [31]. A filter wheel is positioned after the polarizer with bandpass filters (40 nm FWHM, CVI Laser, Albuquerque, New Mexico) centered at 750, 800, 850 nm. After passing each filter, light is being focused onto and captured by a cooled CCD camera (Princeton Instruments CCD-612-TKB, Roper Scientific, Trenton, New Jersey).

The field of view of the image was ∼10 cm with a pixel size ∼400 µm×400 µm. The exposure time of the camera was varied, dependent on skin color, and was set to t = 300 ms for Caucasian skin and t = 400 ms for dark skin. For each patient, a paper mask was placed around the lesion with an imaging area of 6 cm×6 cm, held constant for all patients. For further analysis, the images were cropped in order to only contain the area inside the paper mask or less.

### Principal Component Analysis

Multispectral images were pre-processed to correct for various factors [16]. Images were first corrected for the wavelength-specific camera sensitivity, as well as for the spatial inhomogeneity of the light source. Before further processing, all images were cropped so to only include the area inside the paper mask. Since the images were taken from limbs primarily, the shape of the body part imaged introduces an intensity bias. Also, some of the lesions were nodular, introducing a shape bias as well. To correct for this artifact, we applied a curvature correction algorithm, described previously [32].

In order to extract blood volume and oxygenation values from the intensity data, we applied PCA on the spectral data. PCA extracts the primary components in the data by linearly transforming it onto an orthogonal coordinate system, where the axes correspond to the principal components in the data, which are the eigenvectors of the data. Through an eigenanalysis, the principal components are determined as eigenvectors of the dataset’s covariance matrix and the corresponding eigenvalues refer to the variance that is captured within each eigenvector. The 2D wavelengths images are first transformed into vectors, the mean subtracted, and the covariance matrix calculated and diagonalized:
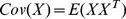
(1)


where 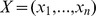 is the zero mean data matrix with pixel vectors 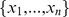. The three eigenvectors 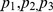 provide the transformed data



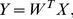
(2)


where 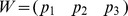. Rearranging the vectors in *Y* into matrices yields again 2D images. Those images represent the projected data along the eigenvector axes, which for simplicity will be referred to as eigenvector images.

We have shown previously [33], [34] that the first two eigenvectors of a three wavelengths reflectance data set using 750 nm, 800 nm, and 850 nm are proportional to the blood volume (eigenvector 1 [EV1]) and blood oxygenation (eigenvector 2 [EV2]) respectively. This finding was based on multi-spectral data from healthy volunteers, with measurements taken on the lower arm. The healthy volunteer population was of diverse background, including skin with high melanin content. We have found that the orientation of EV2 depends on the amount of data content, and that a large data set (>15 sets of 2D images) yields the most reliable results. Using such large data sets we found that the orientation of the obtained eigenvectors of all volunteers was the same with only small deviations of <8 deg (data not shown). Those results suggested that there is one specific eigenvector set, which describes blood volume and blood oxygenation in reflectance data at 750 nm, 800 nm, and 850 nm, independent of melanin content.

Since KS patients data sets were small in comparison (<7 image sets, corresponding to the number of imaging time points), we applied the eigenvectors obtained from healthy skin to KS data. Hence, W in equation (2), was set to:



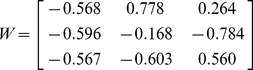
(3)


where each column describes an eigenvector in increasing order from left to right. Each data set for each visit was therefore converted according to equations (1) and (2), with W fixed as shown in (3). It should be pointed out that all measurements on patients were performed on skin areas close to the ones on healthy volunteers (Table 1).

**Table 1:** Patient Characteristics and Response to Treatment.

Patient #	Age (years)	Ethnicity	Imaged Lesion Location	Duration of Therapy (weeks)	Time of Imaged Lesion first Imaging Response (weeks)	Clinical Response of Imaged Lesion to Therapy	Time of Imaged Lesion Clinical Response (weeks)	Time of First Overall Partial Clinical Response (weeks)	Overall Clinical Outcome of Therapy
**1**	48	African American	upper arm	68	56	Responded (Flattened)	56	56	Pathologically confirmed complete remission.
**2**	57	African American	lower arm	79	34	Responded (Flattened)	19	19	Pathologically confirmed complete remission.
**3**	44	Caucasian	lower arm	48	26	Resolved	48	14	Pathologically confirmed complete remission.
**4**	40	Hispanic	lower arm	82	21	Responded (Flattened)	82	21	Pathologically confirmed complete remission.
**5**	35	Hispanic	wrist	81	NA	Stable	NA	NA	Progressive Disease
**6**	52	Caucasian	lower arm	21	2	Responded	21	NA	Progressive Disease*
**7**	31	African American	lower arm	45	NA	Stable	NA	NA	Progressive Disease

In patient 6, the target lesion flattened, but some other flat lesions became nodular and new lesions appeared, so the patient met the overall criteria for progressive disease.

### Correlation to clinical outcome

The primary objective of this study was to investigate if the resulting eigenvector images are correlated with the actual clinical outcome. We hypothesized that the untreated KS lesion shows an increase in blood volume and a decrease in oxygenation in comparison to surrounding uninvolved skin. This is because KS lesions are characterized by disorganized vascular slits filled with relatively static blood. With effective anti-angiogenic therapy, this difference would be expected to become less pronounced over time in responding lesions.

**Figure 2:**
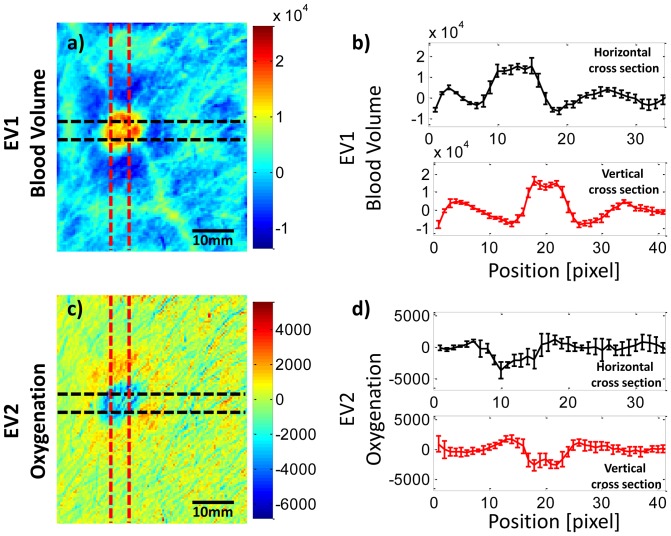
Cross-sections through blood volume and blood oxygenation at baseline in a KS lesion. Cross-sections were chosen to be centered on the lesion, for both blood volume (a) and oxygenation (c). The EV1 and EV2 are depicted in arbitrary units as in Fig. 2. The average of 5 cross sections for blood volume (b) shows the typical increase and for oxygenation (d) shows a decrease inside the lesion area. A surrounding halo of increased oxygenation can be seen, especially to the immediate right of the lesion. The width of the lines in (b) and (d) reflect the range of +/− 1 standard deviation for the 11 cross sections at each point.

For each patient data set, the center of the lesion was selected manually based on guidance from photographs of the lesion, and bands of 11 pixels with the lesion center being in the center were taken in horizontal and vertical directions in the image (Fig. 2). Data was assessed over these 11 adjacent cross-sections, and the average over these 11 cross sections was taken in order to reduce noise. Initially, it was thought that the magnitude of local increases in blood volume in those cross sections, which can be looked at over time, should give a metric of treatment outcome. However, the values in the eigenvector images vary between patients, dependent on the background skin color and variations in the lesion itself. Hence, the eigenvector images are of arbitrary units. Since the eigenvector images are therefore reflective of relative changes, it was impossible to reliably compare the eigenvector image values of different lesions over time and between patients in a quantitative matter. To address this limitation, the standard deviation (SD) over the entire cross sections in the imaging area (area between the dotted lines in Fig. 2) was taken, which gives a metric of smoothness and describes the variation within those parameters. Smoothness was evaluated based on the SD of blood volume and oxygenation across the lesion and surrounding tissue, and for each patient it was normalized by the SD of the baseline (before treatment) cross sections. If the blood volume in the lesion was higher than the surrounding tissue (as it was in all cases), the SD was set as a positive value; otherwise it was set as a negative value. By definition, at the initial baseline evaluation, smoothness is defined as 1 (or -1) for any given patient. This normalized SD with the sign set to reflect the direction of change of the lesion is referred to here as the normalized eigenvalue standard deviation (NESD). Normalizing the SD to the value at baseline allowed us to follow the course of lesions over time and compare the changes between patients. Subsequent smoothness values less than 1 for blood volume were consistent with a change in the direction of normalization of tumor vascularity, and therefore therapeutic effect, whereas subsequent values equal to or greater than 1 were consistent with persistent or worsening abnormal tumor vascularization. We hypothesized that if the treatment were successful, the SD should decrease as the localized variation in these parameters at the lesion area resolved. EV2, a measure of tissue oxygenation, was quantified in a similar manner, and as described above, NESD values were calculated for EV2. For oxygenation, if the lesion had greater oxygenation than the surrounding tissue at a given time, the NESD was given a positive value, and if it had lower oxygenation, it was given a negative value. Hence, the NESD at baseline for each lesion was set at either 1 or -1. It should be pointed out that, since the standard deviation is a metric of noise, or variation, we essentially treated the tumor as variation in the image. Care was taken that the cross sections through the image chosen did not have any other obvious source of measurement variation, i.e. hair or writing on the skin.

We compared these NESD changes to the changes in standard clinical assessment of the studied lesions and the clinical responses of the patients overall. Changes in smoothness of both EV1 and EV2 were compared between those whose lesions responded by standard clinical parameters and those whose lesions did not respond. Imaging response in the target lesion was defined as a reduction in NESD in EV1 by at least 20%.

## Results

### Patient Characteristics and Outcomes

Seven patients with cutaneous KS were studied. Patient demographics and lesion characteristics are shown in Table 1. Patients were treated with one or more of therapeutic approaches in accordance with the primary treatment protocols they were entered on. One patient received the anti-VEGF antibody bevacizumab. The others received a combination of bevacizumab plus cytotoxic chemotherapy with liposomal doxorubicin, followed by bevacizumab alone. For each patient, the response of the imaged “target” lesion by clinical assessment is given together with the overall treatment response. Overall 5 patients were considered to have a clinical response to treatment in the target lesion (Table 1). Of those 5, 4 patients eventually achieved complete overall remissions, confirmed by histopathology of the biopsy of a separate lesion, and are therefore also considered as having a complete response of the target lesion. The 5th patient (patient #6) showed a mixed overall clinical response. In this patient, some lesions, including the imaged lesion, responded and the patient had a sustained decrease in tumor-associated edema. However, some other lesions became nodular and some new lesions appeared, so the patient ultimately met the criteria for progressive disease. It is noteworthy that none of these 5 patients required additional specific therapy for their KS. Two other patients (#5 and #7) had stable disease by both imaging and clinical evaluation of the lesion but had overall progressive disease based on the appearance of new lesions elsewhere. For the purposes of analysis, we therefore divided the patients into 2 groups by the response of the *imaged* lesion: responders (5 patients whose imaged lesion met criteria for at least a partial response based on a decrease in size and/or nodularity) and non-responders (2 patients whose imaged lesion was stable or progressed).

#### Multi-spectral data

**Figure 3:**
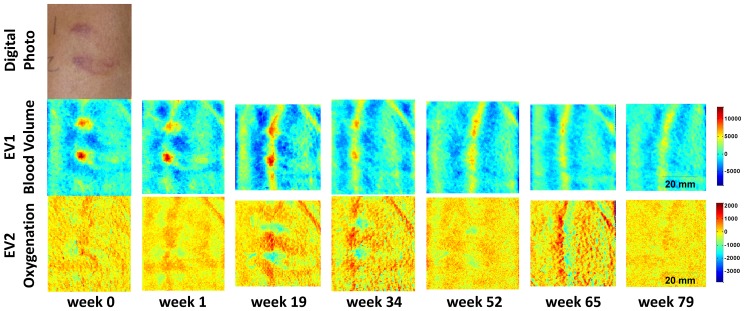
Eigenvector converted blood volume (EV1) and oxygenation (EV2) images. Two adjacent KS lesions over time are shown in the images in arbitrary units of a patient with a confirmed pathological complete response. A digital photograph of the lesions is shown at baseline (week 0).

Each patient’s intensity data was converted into the eigenvector space by equation (3). Fig. 3 shows such a converted data set of one patient (patient #2), whose target lesion achieved a partial response (by criteria of both transformation from nodular to flat and a 50% area reduction) and an overall partial response after 19 weeks of treatment. The patient eventually achieved an overall complete response, confirmed by pathological evaluation of a separate lesion, at week 79. The upper row shows a digital photo of the lesion site at baseline, before treatment. For this patient, two lesions were present in close proximity and were both used for imaging. The middle row shows EV1, which corresponds to blood volume, over time. Both lesions show a local increase in blood volume. The mean value over the image at each time point has been subtracted and all images are on the same color scale, which is in arbitrary units. After 19 weeks of treatment, the lesions are reduced in size and the vessel running through the lesions becomes more visible. By the end of treatment, the localized blood volume in the lesions disappears and only the vessel structure remains and is the most pronounced feature in the image. The lower row of images shows EV2, which corresponds to blood oxygenation, also in arbitrary units. At baseline, the lesions show a small increase in oxygenation (shown by the red coloration) compared to the surrounding tissue. However, at subsequent timepoints, the oxygenation in the lesions is less than the surrounding tissue (shown by the blue coloration), although at the two later timepoints, there appears to be a surrounding region of slightly increased oxygenation.

**Figure 4:**
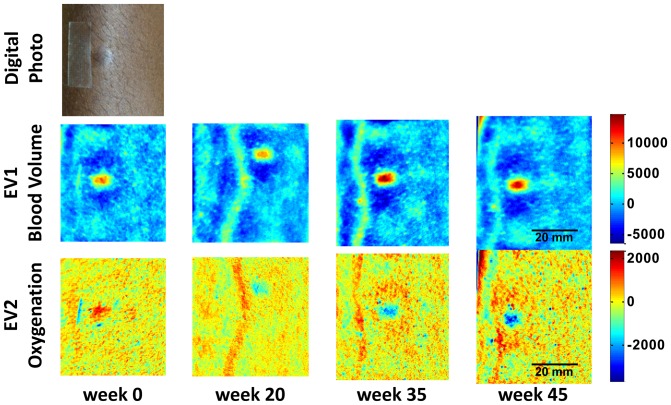
Eigenvector converted blood volume (EV1) and oxygenation (EV2) images. The KS lesion is shown over time in arbitrary units of a patient with progressive disease. A digital photograph of the lesion is shown at baseline (week 0).

In contrast, Fig. 4 shows data from a patient with progressive overall disease after 45 weeks of treatment. Again, a localized increase in blood volume can be seen, which in this case does not disappear, but rather increases over time. Also, a localized decrease in oxygenation over the course of treatment is seen, with the lesion being less oxygenated as the treatment prolongs.

#### Correlation of multi-spectral data to clinical outcome

As described in the previous section, the center of the lesion was identified and the average SD over 11 cross sections through the lesion was calculated in horizontal and vertical directions (Fig. 2). Fig. 2 shows the selection of cross sections for averaging the EV1 (Fig. 2a) and the EV2 (Fig. 2c) images in a typical lesion. The mean values for the 11 cross sections in each direction can be seen for EV1 in Fig. 2b and for EV2 in Fig. 2d, with the width of the lines depicting the range of +/− 1 SD at each point. For this particular patient data, the cross section clearly shows the localized increase for blood volume. Also, this patient shows a localized decrease in oxygenation in the lesion surrounded by a ring shape increase in oxygenation just outside the lesion area. This surrounding increase in oxygenation may correlate with perilesional inflammation.

**Figure 5:**
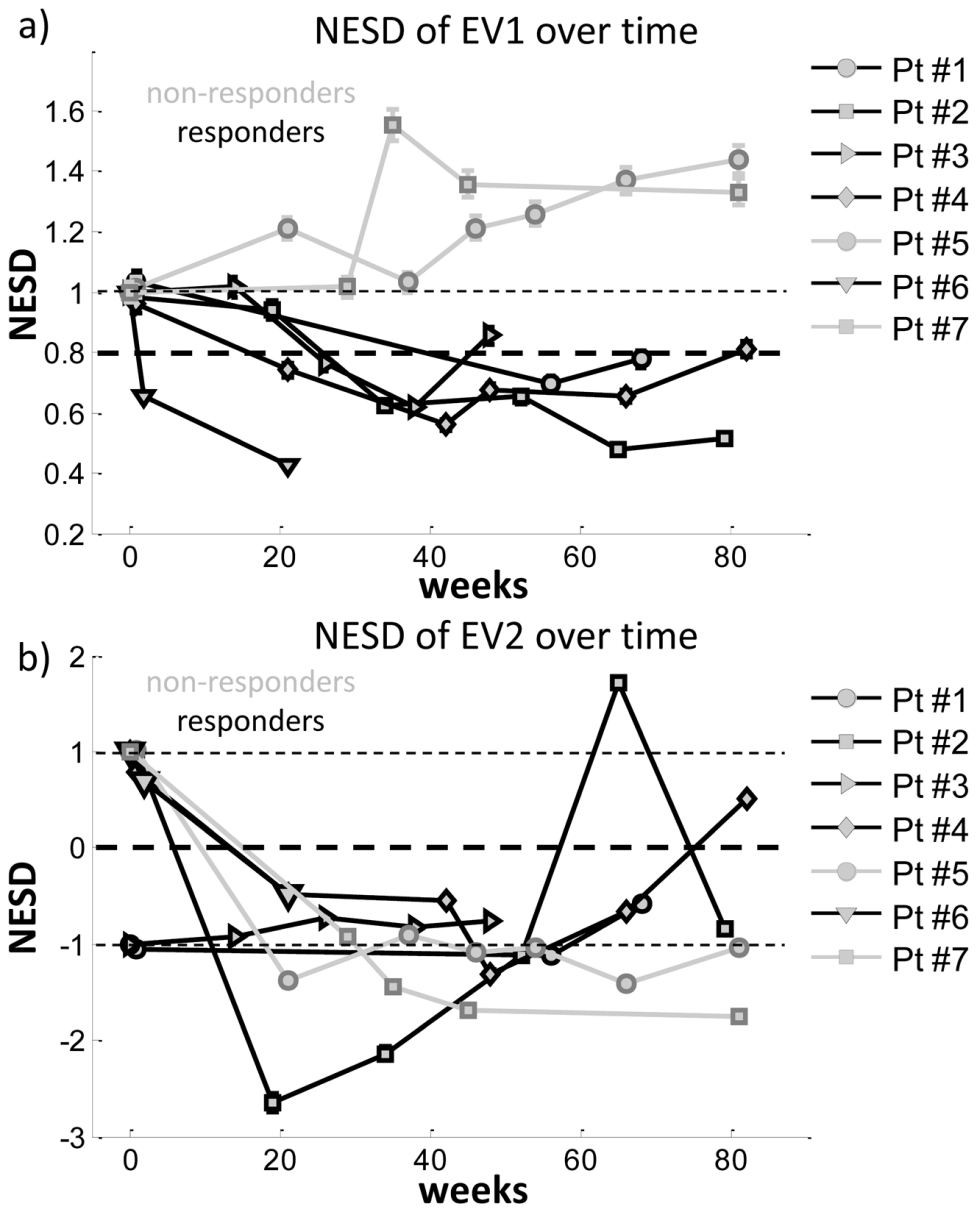
Time courses of NESD for EV1 and EV2. (a) Time course for the NESD of blood volume (EV1) for 5 lesions that showed a clinical response (black) and two lesions that did not show a clinical response (grey). Patients whose lesion responded to treatment show a clear decrease in NESD of blood volume (EV1) over time, whereas those whose lesion progressed show an increase. (b) Time course for the NESD of blood oxygenation (EV2); again the 5 lesions that responded clinically are shown in black, and the 2 lesions that did not respond are shown in grey. The errobars shown correspond to the error on the estimate of the standard deviation. If the errorbar is not visible, the error is smaller than the marker size.

Calculating the overall SD over the mean of the horizontal and vertical cross sections yields 2 values, which we have further averaged, introducing a single value (NESD) for blood volume and another one for oxygenation, which is a descriptive number for the lesion state. As described above, this value is set as a positive number if the overall blood volume (for EV1) or oxygenation (for EV2) of the lesion increased over the surrounding skin, and it is set as negative if the overall blood volume or oxygenation are decreased. By calculating this value for blood volume and oxygenation over time, and normalizing the values to the results at baseline, a time course of normalized ‘image smoothness’ (NESD) was obtained. The time course for NESD of blood volume cross sections can be seen in Fig. 5a. The errobars shown correspond to the error of the estimate of the standard deviation as calculated by Δs = s/(2(n−1))^1/2^, where s is the calculated sample standard deviation (NESD value). All the lesions had increased blood volume compared to the surrounding tissue and the NESD is thus positive. The 5 lesions that had a clinical response are shown in black, and the 2 lesions that did not have a clinical response are shown in grey. The patients with a clinical lesion response to treatment each showed a decrease of NESD of at least 20%, which we have tentatively set as the cutoff for a response, with the maximal decrease being 60%. Using this 20% reduction of EV1 to define an EV1 response, in 4 of the 5 patients who responded, the EV1 response occurred before (and was therefore predictive of) or simultaneous to the clinical response. By contrast, both of the patients whose lesion did not respond clinically showed an increase in NESD of up to 55%.

The timecourse of the NESD for blood oxygenation (EV2) can be seen in Fig. 5b. As with blood volume, each patient data has been normalized by the patient’s baseline measurement, with lesions having increased oxygenation compared to the surrounding skin being given positive values and, and lesions with overall reduced lesion oxygenation being given negative values. At baseline, each lesion was accordingly assigned a value of +1 or −1. Grey lines show the course of this NESD for the lesions with progressive or stable clinical disease (non-responders), while black lines show the NESD for lesions that responded clinically. Interestingly, while 5 of the patients had increased oxygenation at baseline (an NESD of +1), by week 20 all 7 lesions had less oxygenation than the surrounding skin (as evidenced by a negative NESD). It is possible that this change is the result of a decrease in inflammation as a result of the therapy. However, unlike blood volume (EV1), there was no apparent difference between responders and non-responders in this parameter.

## Discussion

In this study we explored diffuse multi-spectral imaging of KS lesions and correlated these imaging results with conventional clinical response assessment to treatment with the goal of finding quantitative objective markers that correlate with, or even predict treatment effectiveness. Evaluation of the blood volume and blood oxygenation concentrations using PCA was performed[33]. All the patients had increased EV1 values (correlating to blood volume) in their lesions at baseline as compared to the surrounding skin. The KS patients were then followed for up to 82 weeks, and the data was divided into two groups, one for patients whose lesion had a clinical response to treatment and the other for patients whose lesion had no response in the imaged lesion. In agreement with our hypothesis that treatment would normalize the blood volume in the lesions, the NESD for blood volume (EV1) decreased in each of the 5 patients whose lesion responded to treatment, while the NESD for blood volume increased in the 2 patients (#5 and #7) whose lesion did not respond (Fig. 5a). It should be noted that patients with KS occasionally have mixed responses to therapy, with some lesions progressing while others resolve. The patient (#6) depicted in Fig. 5a is one such example. This patient had ∼40% decrease in NESD after 1 week and a ∼60% decrease after 21 weeks of treatment and had clinical flattening of the target lesion as well as a clinically significant and measurable improvement in tumor associated edema elsewhere. However, by modified ACTG clinical response criteria, the patient showed variable changes in the lesions with some lesions improving and others initially flattening then later becoming more nodular, and therefore technically met criteria for overall progressive disease. It is not surprising that in this case, the EV1 correlated with the clinical assessment of the lesion imaged, rather than the overall response of the patient, which was driven by progression in other lesions. For future work, it is hypothesized that imaging of multiple lesions would be more reliable than assessment of one lesion for assessing the overall clinical response to therapy.

These results presented here provide evidence that a determination of blood volume by multispectral imaging may be a useful tool to assess and quantify cutaneous KS lesion responses to therapy. This technique is safe and quick to perform, and can thus be easily used to follow lesions over time. Another potential advantage of this technique is that it provides objective and quantifiable data that can supplement clinical assessment disease activity, which can be subject to the observer’s judgment of lesion nodularity and color in relation to surrounding skin. Too few patients, who fit the inclusion criteria, were studied here to permit a formal statistical assessment of the correlation between EV1 and clinical lesion response, and additional patients will need to be studied to further assess this approach. Also, patients were imaged every 3 months. In order to evaluate whether an early distinction between responders and non-responders can be made using NESD, patients will have to be imaged on a more frequent basis early in the course of therapy.

The results for lesion oxygenation were more complex. While we predicted that all patients would have decreased oxygenation (EV2) before therapy, we found that this was not the case; some had decreased oxygenation and some had increased oxygenation. Interestingly, the patients with decreased oxygenation inside the lesion often had a surrounding halo of skin with increased oxygenation. It is quite possible that two opposing effects are present in the lesions. The KS tumor itself is comprised of vascular slits with a disorganized architecture and blood stasis, and these tend to have increased blood volume but also reduced blood flow. At the same time, KS lesions are associated with inflammation that can involve the surrounding tissue, and this can lead to increased blood flow and oxygenation. It is noteworthy that after therapy with an anti-angiogenic agent with or without chemotherapy, tissue oxygenation in the lesions fell below that of the surrounding tissue in all cases. It is possible that bevacizumab-based therapy, even when not successful, decreases tumor oxygenation by reducing KS cytokine production and inflammation.

The results of this study suggest that multispectral imaging may have utility in the assessment of KS lesions and perhaps other cutaneous tumor lesions as well. In particular, it shows promise as a means of evaluating hemoglobin related changes in assessed lesions during therapy. At the present time, clinical assessment of KS lesions involves demonstrating that a nodular lesion becomes flat, that a lesion decreases in area (usually by 50%), or that the lesion resolves completely. In practical terms, the most likely parameter to show benefit is the flattening of a nodular lesion, and assessment of this is subjective and susceptible to variation among evaluators. Also, lesions usually do not shrink in size, and when they resolve can leave some residual pigmentation from extravasated blood, compounding the difficulties in assessing clinical responses. As seen in the present study, evaluation of blood volume by multispectral imaging and PCA is simple to perform, non-invasive, and correlates with clinical changes. The results here also suggest that changes of 20% can be reliably detected. In most cases, using the 20% threshold, responses in a lesion were documented by imaging before they were documented using clinical criteria. It should be pointed out again that we have imaged the patients every three months. The time points of imaged lesion response reported in table 1 were based on when we imaged the patients. It is anticipated that an earlier response would have been seen if the patients were to be imaged in shorter intervals. Additional studies will be needed to assess the reproducibility of these results, determine the optimal decrease use to define a response, and compare these results to clinical assessment in additional patients so as to develop a statistical assessment of its utility. These intriguing results suggest further evaluation of this imaging modality in patients with KS is warranted to establish its utility as a biomarker that is objective and may detect responses before they meet standard clinical criteria or be used as an alternative to biopsy to assess disease activity in equivocal residual lesions in treated KS. Furthermore, additional studies of the effects on blood oxygenation may yield insights into the pathophysiology of KS lesions at baseline and during therapy.

## Summary

When assessing patients with KS and other cutaneous diseases, it is useful to be able to perform real time assessments of the lesion and its metabolic state. Diffuse multi-spectral imaging with PCA analysis was assessed as a potential tool to reconstruct blood volume and oxygenation concentrations. KS patients were imaged over the course of treatment and were divided into two groups, responders and non-responders. We found that the uniformity of blood volume throughout the image can be used as a reliable marker for the status of the lesions and treatment efficacy. The uniformity of blood volume as assessed by the NESD of a cross section through the lesion was found to correlate with clinical determination of lesion response. Also, in 4 of 5 cases in which the imaged lesion responded, a 20% decrease in blood volume could be detected before a subsequent clinical lesion response.
